# Individualized Proteogenomics Reveals the Mutational Landscape of Melanoma Patients in Response to Immunotherapy

**DOI:** 10.3390/cancers13215411

**Published:** 2021-10-28

**Authors:** Marisa Schmitt, Tobias Sinnberg, Heike Niessner, Andrea Forschner, Claus Garbe, Boris Macek, Nicolas C. Nalpas

**Affiliations:** 1 Quantitative Proteomics, University of Tübingen, 72074 Tübingen, Germany; marisa.schmitt@uni-tuebingen.de; 2 Division of Dermatooncology, University of Tübingen, 72074 Tübingen, Germany; Tobias.Sinnberg@med.uni-tuebingen.de (T.S.); Heike.Niessner@med.uni-tuebingen.de (H.N.); andrea.forschner@med.uni-tuebingen.de (A.F.); claus.garbe@med.uni-tuebingen.de (C.G.); 3 Cluster of Excellence iFIT (EXC 2180) “Image-Guided and Functionally Instructed Tumor Therapies”, University of Tuebingen, 72074 Tübingen, Germany

**Keywords:** proteogenomics, melanoma, mass spectrometry, immunotherapy, whole exome sequencing

## Abstract

**Simple Summary:**

Melanoma is the most aggressive form of skin cancer, with a rapidly increasing incidence rate. Due to ineffective treatment options in the late stage melanoma, patients have an overall poor prognosis. Over the last decades, the role of the immune system in the control of tumor progression has been established and immune checkpoint inhibitors (ICi) have shown remarkable clinical activity. While current trials suggest durable responses in patient under ICi therapy, there is increasing evidence pointing towards existence of innate and acquired resistance to ICi therapy; and it is now clear that personalized medicine will be critical for effective patient therapy. Proteogenomics is a powerful tool to study the mode of action of disease-associated mutations at the genome, transcriptome, proteome and PTM level. Here, we applied a proteogenomic workflow to study melanoma samples from human tumors. Such workflow may be applicable to other patient-derived samples and different cancer types.

**Abstract:**

Immune checkpoint inhibitors are used to restore or augment antitumor immune responses and show great promise in the treatment of melanoma and other types of cancers. However, only a small percentage of patients are fully responsive to immune checkpoint inhibition, mostly due to tumor heterogeneity and primary resistance to therapy. Both of these features are largely driven by the accumulation of patient-specific mutations, pointing to the need for personalized approaches in diagnostics and immunotherapy. Proteogenomics integrates patient-specific genomic and proteomic data to study cancer development, tumor heterogeneity and resistance mechanisms. Using this approach, we characterized the mutational landscape of four clinical melanoma patients. This enabled the quantification of hundreds of sample-specific amino acid variants, among them many that were previously not reported in melanoma. Changes in abundance at the protein and phosphorylation site levels revealed patient-specific over-represented pathways, notably linked to melanoma development (MAPK1 activation) or immunotherapy (NLRP1 inflammasome). Personalized data integration resulted in the prediction of protein drug targets, such as the drugs vandetanib and bosutinib, which were experimentally validated and led to a reduction in the viability of tumor cells. Our study emphasizes the potential of proteogenomic approaches to study personalized mutational landscapes, signaling networks and therapy options.

## 1. Introduction

One of the hallmarks of cancer cells is the accumulation of mutations and malignant melanoma is a type of cancer with the highest frequency of somatic mutations [1]. Mutations of key signaling pathways in malignant melanoma are associated with poor clinical outcomes [2]. For example, up to 50% of cutaneous melanomas harbor non-synonymous mutations in the kinase BRAF [3]. The predominant BRAF mutation (V600E) is found within the kinase domain [4] and leads to the constitutive activation of downstream signaling in cancer cells [3,5]. Targeted inhibition of the mutated BRAF kinase with selective inhibitors such as vemurafenib, dabrafenib or encorafenib (BRAFi) results in a reduction in activity in the MAPK pathway [5]. However, almost all patients rapidly develop resistance to BRAFi monotherapy, which calls for the development of novel therapeutic options [2].

Over the last decades, the role of the immune system in the control of tumor progression has been established and new immunotherapeutic targets have shown remarkable clinical activity. The reagents nivolumab and ipilimumab are immune checkpoint antibodies targeting PD-1 (programmed cell death-1) and CTLA-4 (cytotoxic T lymphocyte-antigen-4) receptors [6,7]. PD-1 and CTLA-4 are co-inhibitory T cell receptors and act as negative regulatory receptors that block T cell activation and induce immune tolerance [8,9]. Subsequently, obstruction of these receptors with antibodies has demonstrated tumor rejection and a significant prolongation of the survival of the melanoma patient. However, only a minority of patients responded to ipilimumab and many patients developed immune-related toxicities [10,11]. The response or resistance to the immune checkpoint blockade is determined by complex and multiple mechanisms, such as the heterogeneity in the immune response across tumors, the tumor microenvironment and the varying tumor immunogenicity [12]. The clinical response to immune checkpoint inhibitors and resistance is often associated with a high mutational load and the number of expressed tumors neoantigens leading to antitumor immunity [13,14]. Several studies have shown that deficiencies in antigen presentation and the down-regulation of MHC class I (MHC-I) play a role in immune checkpoint resistance [15,16,17]. Besides the mutation in β2-microtubulin, the loss of the JAK-STAT pathway results in an acquired resistance due to the down-regulation of MHC-1 [18,19]. Additionally, classic oncologic pathways such as the MAPK, PI3K-AKT or WNT/β-catenin pathways can regulate immune responses by influencing the tumor’s microenvironment. Alterations in the MAPK pathway may lead to increased expression of VEGF, a vascular endothelial growth factor, and other inhibitory cytokines, thus mediating the evasion of tumor cells [20,21]. Constitutive activation of the PI3K-AKT pathway due to loss of PTEN has been associated with resistance to PD-1 therapy and the decreased overall survival of patients with leiomyosarcoma [22,23]. The majority of these studies were performed at the genomic and transcriptomic level. Transcriptomic signatures of cytosolic markers and immune-related genes could predict the clinical response and outcome of patients with different therapies [24]. Melanoma sub-populations showed a heterogeneity in the transcriptional processes, for example, the CDK4 and CDK6 regulated pathways were linked to resistance mechanisms in the non-responder’s cells studied by single cell RNA sequencing. In a quantitative proteomic screen, Harel et al. compared clinical melanoma samples treated with either tumor-infiltrating lymphocyte (TIL) or anti PD-1 immunotherapy and showed an association between higher lipid metabolism and response to immunotherapy [25].

Standard proteomic approaches identify peptides and proteins by matching MS/MS spectra against protein databases derived from public repositories (e.g., UniProt) that are not individualized. By combining nucleotide sequencing and MS technologies, it is possible to simultaneously study and integrate DNA sequences, RNA expression and splicing, protein isoform abundance and post-translational modifications (PTMs) in a patient-specific (personalized) fashion. Genomic alterations due to non-synonymous single nucleotide variants (nsSNVs), insertions or deletions (InDels) of nucleotides, frameshifts and alternate splicing variants can alter the cellular function at the protein level by modulating its abundance, localization and protein–protein interaction [26,27]. Clinical data have shown that oncogenic targets are aberrantly post-translationally modified during tumorigenesis and might be relevant as therapeutic targets [4]. The most prominent protein modification is phosphorylation, which is abnormally activated during tumorigenesis and may propagate dysregulated signals and cellular functions [28,29]. However, such alterations affecting the modification level in signaling molecules can also be benign and insignificant.

Here, we used melanoma tissue from human tumors and matching patient-derived xenografts to study the patient-specific mutational landscape in response to immunotherapy. We reconstructed the signaling transduction network specific to individual patients using their corresponding genomics, proteomics and PTMs datasets.

## 2. Results

### 2.1. The Mutational Landscape of Melanoma Patients in Response to Immunotherapy

**Figure 1:**
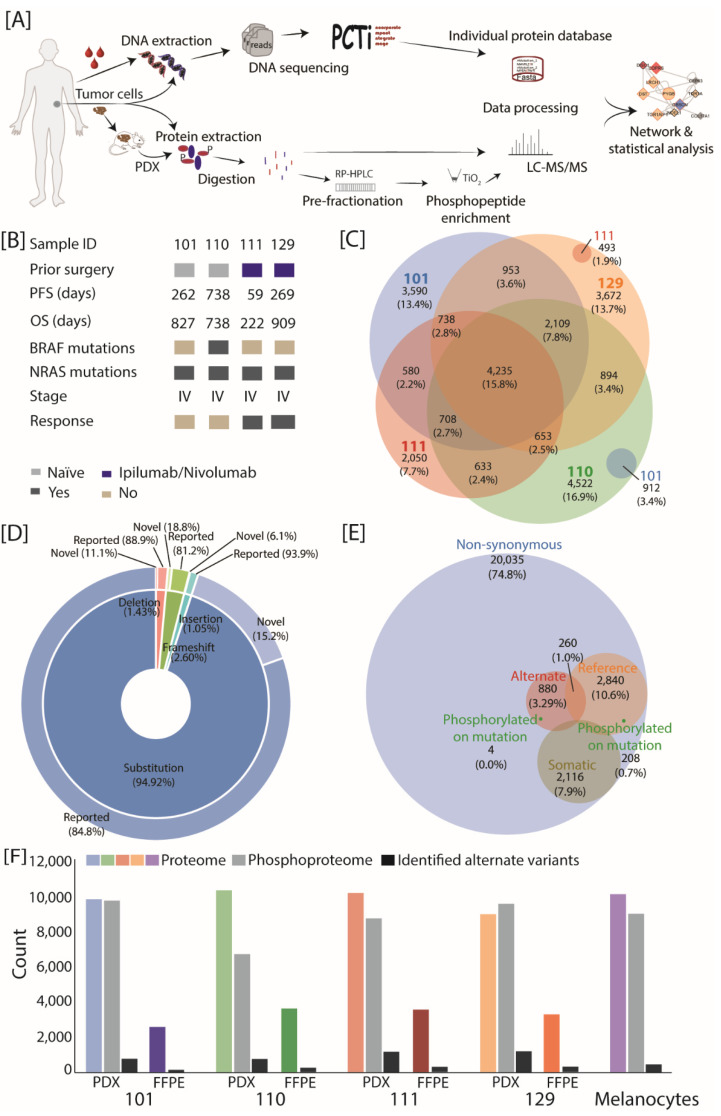
The mutational landscape of melanoma patients in response to immunotherapy. (**A**) Schematic overview of the proteogenomic workflow. Whole blood and tumor tissue of four patients were used in this study. Metastatic tumor tissue was injected into an immune-deficient mouse to generate patient-derived xenografts (PDX). For whole exome sequencing, DNA was extracted from whole blood and metastatic tissue and sequenced on an Illumina sequencing instrument. Individualized protein databases and impact files were generated with an in-house bioinformatic pipeline. For the proteomic workflow, FFPE specimens from the same tissue as well as patient-derived xenografts tissue were used. Cells were lysed and proteins were digested using trypsin. The resulting peptide mixture from the PDX material was fractionated using an off-line RP HPLC operated at a high pH. Fractions were pooled and measured directly or applied to phosphopeptide enrichment using titanium dioxide (TiO_2_) prior to LC-MS/MS. MS raw data was processed with MaxQuant software and analysed by PCTi. (**B**) Clinical information of analyzed samples including the administered therapy, the progression-free survival (PFS), overall survival (OS), detection of variants in key oncogenes, cancer stage and clinical outcome. The PFS and OS were calculated based on the start of therapy and the numbers indicate the days after therapy started. (**C**) Overlap of non-synonymous nucleotide variants identified by WES of four melanoma patients (tumor tissue and blood). (**D**) Inner donut depicts the type of all non-synonymous nucleotide variants identified by WES including substitution, insertions, deletions and frameshifts. Outer donut represents the proportion of novel nucleotide variants identified in this study. (**E**) Overlap in identified nucleotide variants (from all patients) between WES-identified non-synonymous variants (blue), WES-identified non-synonymous somatic variants (brown), MS-identified reference variant peptides (orange), MS-identified alternate variant peptides (red) and MS-identified phosphorylated on variant site peptides (green). Numbers correspond to the size of the set or the percentage of the total. To allow a comparison between WES and MS identification, variants were counted at the nucleotide level (avoiding redundancy from protein isoforms). (**F**) Identified protein groups and variants by MS for each patient and sample type (PDX and FFPE) and the number of phosphorylation sites identified in the PDX samples. Identified alternate variant protein isoforms per patient are shown in black.

In order to identify the signatures and cellular mechanisms of immunotherapy response, we analyzed matching clinical samples including blood, formalin-fixed paraffin-embedded (FFPE) tumor tissue and patient-derived xenografts (PDX) from four patients (Figure 1A). Two of the analyzed patients were naïve (no treatment at the time point of sampling, patient IDs 101 and 110) and two patients were treated with immune checkpoint inhibitors (ICi) nivolumab and ipilimumab at the time point of surgery (patient IDs 111 and 129, Figure 1B and Table S1). The progression-free survival (PFS) and overall survival (OS) were calculated based on the start of therapy and differed in all patients. The patient under therapy with the ID 111 showed a shorter PFS and OS compared to others. Only one patient presented the well characterized BRAF^V600E^ mutation; however, all patients showed NRAS mutations at different sites (G12V, Q61R, A146T, F156L), which is the second-most mutated gene in melanoma [30].

For proteogenomic analysis, we performed whole exome sequencing (WES) from snap-frozen tumor tissue and matching blood samples, allowing the detection of germline and somatic nucleotide variants (Figure 1A). The sequencing depth was similar across the samples (Figure S1A). Among all the non-synonymous nucleotide variants detected by WES (ca. 23,000), more than half were unique to one of the four patients, whereas only 15.8% were identified in all four patients (Figure 1C). The number and type of nucleotide variants detected by WES were similar across all four patients (Figure 1C,D); the vast majority were substitutions, most of which have been previously reported in either dbSNP or Cosmic databases (84.8%). Comparison of the WES analysis of blood and tissue samples enabled us to distinguish between germline and somatic nucleotide variants (Figure 1E), which were present in an approximate 1:10 ratio.

The identified non-synonymous variants were incorporated into the corresponding protein sequence, thus providing protein sequence databases that were individualized for each patient (Table S1). The proteomic (PDX and FFPE) and phosphoproteomic (only PDX) datasets of each sample were processed against the human reference and individualized protein databases in order to identify reference (i.e., corresponds to an amino acid sequence from the reference database) and alternate variant peptides (i.e., corresponds to a sequence containing an amino acid variant). The mouse database was also included during processing to assess contamination with mouse proteins (i.e., relevant for PDX samples), but exclusively murine identifications were ignored for subsequent analyses. To enable a patient-specific comparative analysis, we also performed proteomics and phosphoproteomics of a generic healthy melanocyte sample. The rationale behind this experimental design spans from the lack of patient-specific healthy tissue to compare against patient tumor material, thus a common healthy standard sample was used.

Overall in the PDX samples, we identified over 9500 proteins and 120,000 sequence-specific peptides per sample that were annotated as human (Figure 1F, Table S2). The phosphoproteomic analyses (PDX samples) identified over 9000 phosphorylation sites for patient IDs 101, 111 and 129, while over 5000 phosphorylation sites were detected for patient ID 110. The patient IDs 110 and 111 showed the highest proportion of alternate variant protein isoforms compared to the overall identified proteins. Interestingly, the scores and intensities of identified peptides were similar between the reference and alternate variant peptides, highlighting the overall good quality of the MS-identifications (Figure S1B,C). In agreement with the WES results, a majority of MS-identified alternate variant peptides were patient-specific (Figure S1D). We identified approximately 125 alternate variant peptides in patient IDs 101 and 129, and over 300 in patient IDs 110 and 111.

In the FFPE samples, the global proteome revealed more modest protein identification (between 2000 and 4000 per patient), due to limited material amounts (Figure 1F). A comparison between the sample types revealed that between 85% and 95% of proteins quantified in FFPE were also quantified in PDX (Figure S1E). A correlation analysis between these commonly quantified proteins displayed decent positive correlation levels (Spearman rho between 0.5 and 0.75), which supports the use of PDX as a model to study cancer progression. Overlap proportion was also consistent when only the alternate variant protein isoforms were considered (lowest overlap in patient ID 101, highest in patient ID 111). A principal component analysis (Figure S1F) also confirmed the clustering of samples (PDX and FFPE) per patient IDs based on component two and three (explaining 17.8% of variance). Taken together, our results highlight the importance of individualized approaches in order to investigate patient-specific tumors and the potential of PDX for in-depth proteomic investigation.

### 2.2. A Comparison of Tumor Cells against Melanocytes Highlights Patient-Specific Signaling Pathways

**Figure 2:**
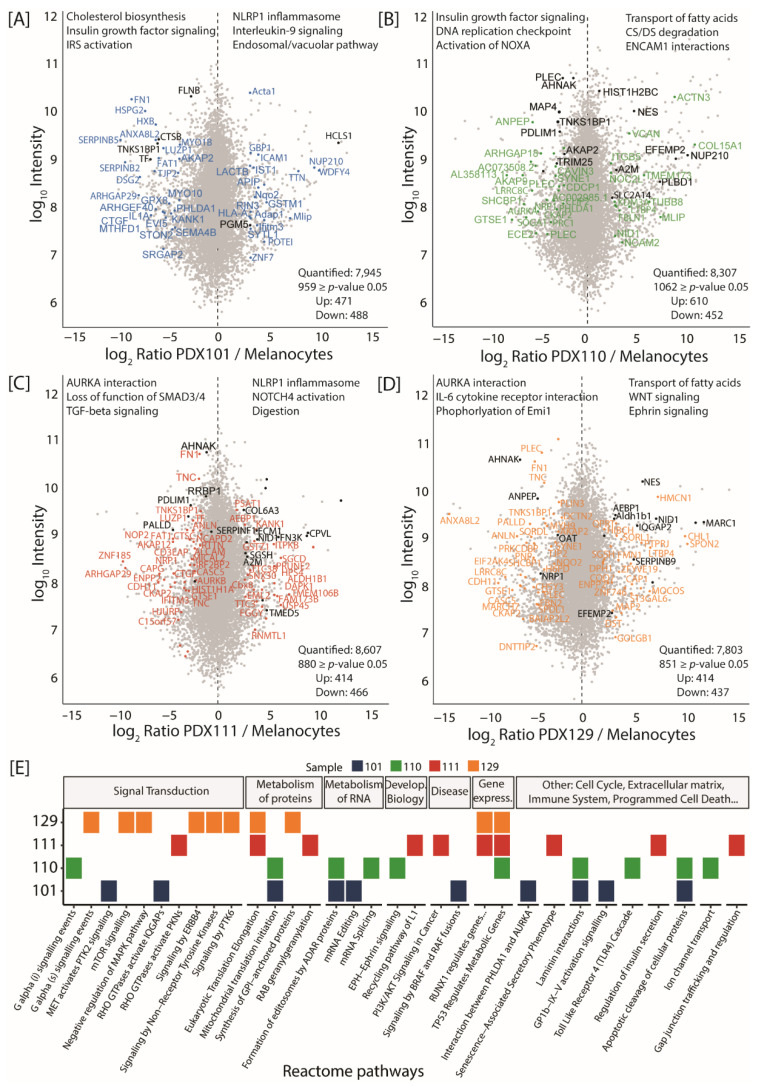
Comparison of tumor cells against melanocytes highlights patient-specific signaling pathways. (**A**–**D**) Scatter plot of log_2_-transformed ratios for proteins quantified in the PDX samples versus melanocytes for patient IDs 101 (**A**), 110 (**B**), 111 (**C**) and 129 (**D**). Significant proteins containing identified alternate peptides are marked in the respective color (significance B, *p*-value ≤ 0.05). Proteins marked in black were also identified in the corresponding FFPE material for each patient ID. The top 3 over-represented Reactome pathways based on all significantly up- or down regulated proteins are depicted in the upper part of each panel (Fisher-Exact test, *p*-value ≤ 0.2). (**E**) Heatmap of over-represented pathways within each patient based on proteins containing alternate variant peptides. Results are based on the Fisher-Exact test (*p*-value ≤ 0.2). Color-coding indicates if a specific pathway was significantly over-represented in patient IDs 101 (blue), 110 (green), 111 (red) and 129 (orange).

Subsequently, we compared the global proteome from each patient (using both PDX and FFPE samples) against a generic healthy melanocyte sample in order to investigate patient-specific protein changes. This led to the identification of 959, 1062, 880 and 851 proteins with significant changes in abundance (*p*-value ≤ 0.05) in patient IDs 101, 110, 111 and 129, respectively (Figure 2A). Significantly up-regulated proteins were over-represented in immune-related pathways such as “interleukin-9 signaling” or “NLRP1 inflammasome” for patient ID 101 (Figure 2A). In both naïve patients (ID 101 and 110), proteins related to “insulin growth factor signaling” were down-regulated in comparison to melanocytes (Figure 2A,B), whereas in both ICi-treated patients (ID 111 and 129) several “AURKA interactors” were down-regulated (Figure 2C,D). Up-regulated proteins in both ICi-treated patients differed; however, most pathways could be linked to immunotherapy. Notably, “NLRP1 inflammasome” and “NOTCH4 activation” were over-represented in patient ID 111, whereas “WNT and Ephrin signaling” were over-represented in patient ID 129. We also compared the global phosphoproteome from each patient (using only PDX samples) against the melanocytes (Figure S2A–D). For all four patients, the “MAPK1 or MAPK1/3 activation” pathways—known for their pivotal involvement in melanoma—were enriched in significantly up-regulated phosphorylation sites.

Next, we focused on proteins harboring MS-identified alternate variant peptides. Their over-representation among KEGG or Reactome pathways revealed “signatures” specific to each patient (Figure 2E). There seemed to be a more consistent overlap in over-represented pathways among either ICi-treated or naïve patients, whereas the overlap was nearly non-existent between ICi-treated and naïve patients. For example, the pathways related to transcriptional regulation by RUNX1 and eukaryotic translation elongation were enriched in both ICi-treated patients. On the contrary, naïve patients showed an over-representation for pathways such as the apoptotic cleavage of cellular proteins, laminin interactions, mitochondrial translation initiation and formation of editosomes by ADAR proteins. Among the many patient-specific pathways were “Rho GTPase activate protein kinases (PKNs)” and “PI3K/Akt signaling in cancer” (in patient ID 111); “mTOR signaling” as well as “signaling by ERBB4 or PTK6” (in patient ID 129); “TLR4 cascade” and “G alpha signaling events” (in patient ID 110); “signaling by BRAF and RAF fusion” as well as “RHO GTPases activate IQGAPs” (in patient ID 101). Notably, the pathway “TP53 regulates metabolic genes” was enriched in three patients, ID 110, 111 and 129. These findings demonstrate the use of the personalized proteogenomic approach to characterize patient-specific regulated proteins, as well as patient-specific variants and their accumulation in key pathways.

### 2.3. Integration of Genomics, Proteomics and Drug Database Prioritizes Actionable Targets

**Figure 3:**
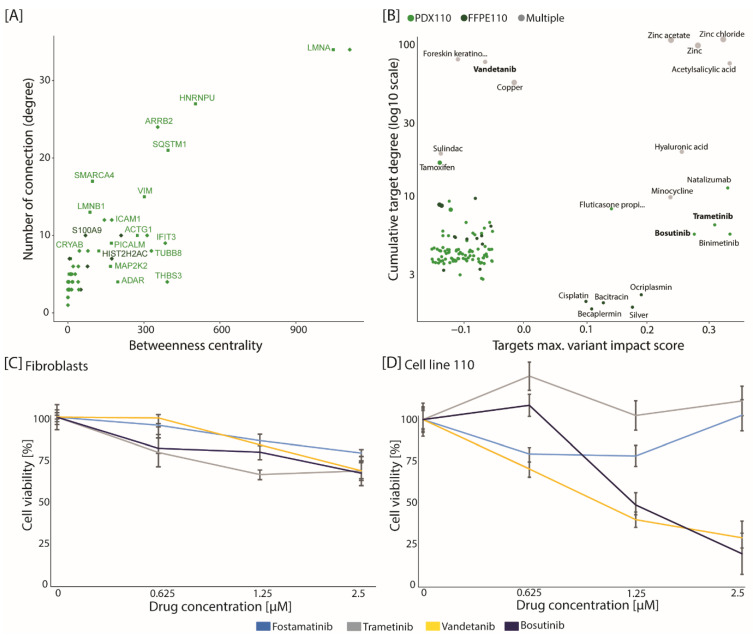
Integration of genomics, proteomics and drug database prioritizes actionable targets. (**A**) The interaction signaling network for patient ID 110 was generated based on list of significantly regulated proteins (diamond) and phosphorylation sites (square). This schematic displays the distribution of nodes in function of their betweenness centrality and number of connections. Only the top 200 entries are displayed (ranked based on their interaction degree). Entries are colored based on whether they were up-regulated in PDX (light green) or FFPE (dark green). (**B**) The drugs, interacting with entries from the interaction signaling network of patient ID 110, are displayed based on their targets’ maximum variant impact score and how many connections their targets had. Color-coding corresponds to whether all of the drug targets were specific for PDX (light green), FFPE (dark green) or common to both sample types (grey). (**C**,**D**) Cell viability assay for fibroblasts (**C**) and cell line of patient ID 110 (**D**) treated with either fostamatinib (blue), trametinib (grey), vandetanib (yellow) or bosutinib (dark blue). Cells were cultured for 24 h, and then treated with the depicted drugs at the indicated concentrations (0, 0.635, 1.25 and 2.5 µM) or DMSO as the control. Cell viability was determined by MTS assay 96 h later. Results expressed as a percentage of the control represent the mean of six replicates. The error bar represents the standard deviations of replicates.

To define actionable protein targets, we integrated the significantly changing proteins and phosphorylation sites into several patient-specific protein–protein interaction networks. Several entries were further emphasized due to their high betweenness centrality and degrees, as well as their targetability by a drug (Figure 3A, Figure S3A, Table S3). For patient ID 110, this notably included MAP2K2, KIT, VEGFA, A2M, ICAM1 and PLA2G4A, all of which are involved in cancer development.

This approach resulted in the prediction of drugs that could impact the patient-specific perturbed signaling network (Figure S3A). While drugs could be predicted for each patient, the patient ID 110 was associated with twice as many drugs (107 potential drugs) as any other patient (Figure S3B). Between 10% and 30% of the patient-specific predicted drugs were also known oncotherapeutics and thus of high interest. The majority of predicted drugs were specific for each patient and the overlap in patient-predicted drugs was relatively modest—only 10 potential drugs were shared across all four patients (Figure S3C, Table S3). We then prioritized the potential drugs by focusing on known oncotherapy and by maximizing either the number of degrees their targets had with the rest of the signaling network or the variant impact score of their protein targets (Figure 3B). For patient ID 110, the prioritized onco-therapies included drugs with a high number of degrees, such as vandetanib or tamoxifen, and drugs whose target proteins had a high impact variant, such as trametinib, bosutinib or binimetinib. These drugs are inhibitors of EGFR and VEGFA (vandetanib), PRKCD and PRKCE (tamoxifen), as well as MAP2K1 and 2 (e.g., trametinib, bosutinib, binimetinib). While all prioritized therapies would require experimental validation, we focused on only three drugs, i.e., vandetanib, bosutinib and trametinib. Remarkably, the predicted drugs vandetanib and bosutinib showed a reduced cell viability in cells generated from the tumor material of patient ID 110 (Figure 3D), whereas no effect on cell viability was observed in control fibroblast cells (Figure 3C). The variant sites for MAP2K1 and KIT were confirmed by sanger sequencing, whereas for EGFR no variant was observed (Figure S3D). Overall, the network modeling approach allows the prediction of patient-specific therapies and demonstrates the potential of precision medicine.

### 2.4. Differential Protein Expression between Naïve and ICi-Treated Patients

**Figure 4:**
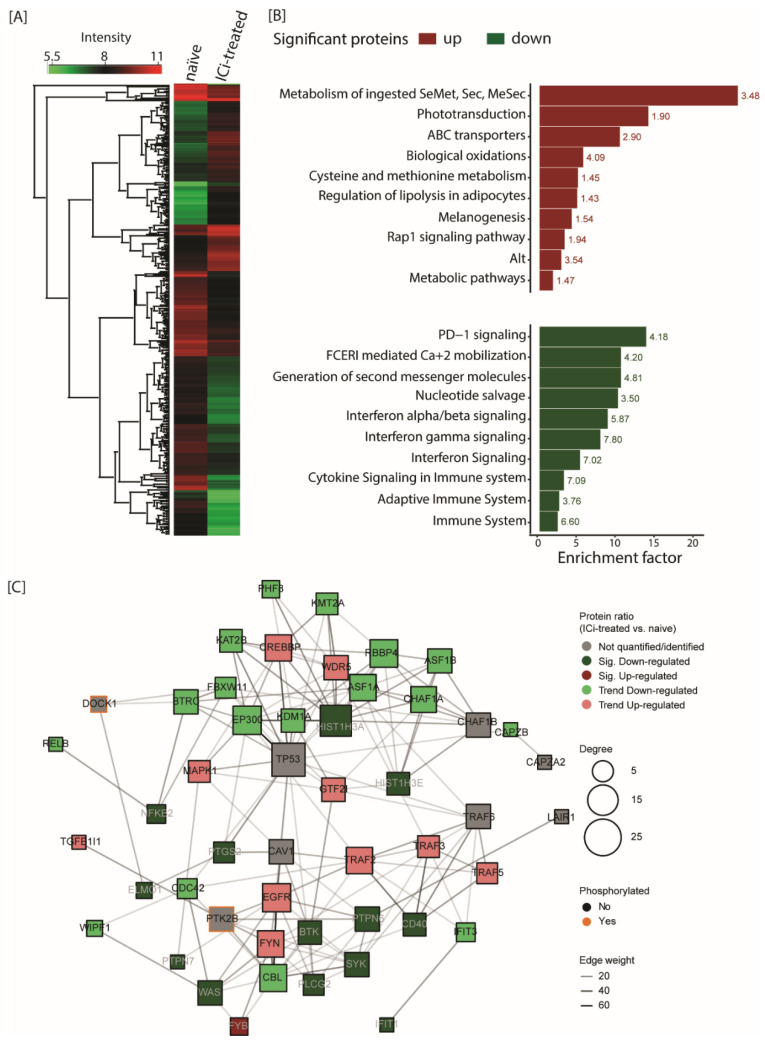
Differential protein expression between naïve and ICi-treated patients. (**A**) Heat map of significantly regulated proteins between naïve and ICi-treated patients (Sig. B, FDR ≤ 0.05). Color code depicts log_10_-transformed IBAQ intensities of proteins for each group. (**B**) Pathway over-representation of significantly regulated proteins between naïve and ICi-treated patients (Sig. B with FDR ≤ 0.05; Fisher-Exact test with FDR ≤ 0.02). Pathways over-represented based on up- and down-regulated proteins are displayed in red and green, respectively. The text on the right of each bar corresponds to the over-representation of the −log_10_ *p*-value. (**C**) Interaction network of immune related proteins significantly changed between ICi-treated and naïve patients, as well as their direct protein interactors. Only the top 50 entries are displayed (ranked based on their interaction degree). Entries are colored based on their direction of regulation between ICi-treated and naïve patients; i.e., not quantified (grey), down-regulation trend (light green), significant down-regulation (dark green), up-regulation trend (light red) and significant up-regulation (dark red). Entries that were also found to be phosphorylated are displayed with an orange stroke. Node size is proportional to the node number of connections (degree).

Next, we compared the naïve and ICi-treated patients (PDX) based on the global proteome. In total, we identified 436 proteins that were significantly regulated between naïve (IDs 101 and 110) and ICi-treated (IDs 111 and 129) patients (FDR ≤ 0.05) (Figure 4A and Table S4). Up to 10% of the significantly regulated proteins showed a possible gain or loss of a S/T/Y site and 17.2% were identified to be phosphorylated.

Pathway over-representation of significantly regulated proteins between naïve and ICi-treated patients revealed that significantly up-regulated proteins were enriched in the “metabolism of ingested SeMet and Sec”, “Melanogenesis” and “RAP1 signaling” pathways (Figure 4B, Table S4). Notably, down-regulated proteins were related to PD-1/PD-L1 and cytokine signaling. This might be due to the fact that both ICi-treated models were isolated from metastases that had grown upon anti-PD-1-based ICi and thereby can be considered as resistant lesions. Lack of PD-L1 expression as well as the loss of IFNg signaling are long-known resistance mechanisms to ICi therapy addressing PD-1/PD-L1 [31]. Interestingly, 86 of the 436 regulated proteins harbored an alternate variant peptide (Figure S4A–C). Over-representation analysis based on the regulated proteins harboring an alternate variant peptide also revealed pathways important in the context of melanoma development, such as “interferon signaling” and “extracellular matrix degradation/organization” pathways.

To investigate the differing immune system response between the naïve (IDs 101 and 110) and ICi-treated (IDs 111 and 129) patients, we generated a signaling network of significantly changing immune-related proteins (FDR ≤ 0.05) together with their direct interactors (Figure 4C, Table S4). This network was derived mostly from 13 significantly down-regulated proteins in ICi-treated versus naïve patients, compared to only one up-regulated protein. Among these, the tumor necrosis factor receptor superfamily member 5 (CD40), the tyrosine-protein kinase SYK or the nuclear factor NF-kappa-B p100 subunit (NFKB2) proteins were all down-regulated and are well-known for their involvement within immune pathways. Altogether, this suggests a down-regulation of the immune response in ICi-treated patients, which may be driven by a selection for ICi-resistant cancer cells [18].

## 3. Discussion

Here we present the individualized proteogenomic landscape of four melanoma patients in response to immunotherapy. This study is, to our knowledge, the first integrative proteogenomic analyses of melanoma tumor tissue and matching PDX in response to immunotherapy. Malignant melanoma has predominantly been studied by genomics and transcriptomics, and more recently by proteomics [32,33]. As the majority of drugs target proteins, proteomics allows extensive and quantitative surveys of the global proteome in order to select targeted treatment and predict drug response in tumor therapy. However, proteomics is not individualized and publicly available databases do not contain cancer- and sample-specific variants. Several genomics and transcriptomic studies revealed the mutational landscape and heterogeneity of melanoma cases [34,35,36]. A recent quantitative proteomic screen of a melanoma patient’s tumors in response to immunotherapy revealed the link between lipid metabolism and the response to immunotherapy [25]. Lobas et al. used a proteogenomic approach to study eight melanoma cell lines; their analysis allowed discrimination between the specific cell lines based on their variant peptide profiles [37].

Importantly, our dataset was generated from four patients, which certainly cannot recapitulate the patient tumor heterogeneity observed in large cohort studies [38,39,40,41]. Therefore, our study did not aim to characterize the pathways generally involved in melanoma development and resistance, but focused on showcasing the application of proteogenomics in the context of precision oncology [42]. Here, 15.2% of the identified nucleotide variants were not reported previously, indicating a high variability in the mutational landscapes of cancer patients. We identified a number of shared as well as sample-specific alternate variant peptides by whole exome sequencing and mass spectrometry. The identifications are in the same range or even better in comparison to other proteogenomic datasets of human cancer tissue [38,43,44]. The detected alternate variant peptides were of a high quality based on the MaxQuant-derived score, which was similar to the reference variant peptides. We also did not observe a change in intensity or score distribution between the reference and alternate variant peptides, which would have been indicative of reduced quality.

Here, the proteogenomic signatures of PDX confirm most findings from melanoma cancer patients. Thus, PDX samples, which are tumor tissues closely resembling the clinical lesions, can serve as models to study the mutational landscape of cancer. PDXs overcome several limitations over the use of monolayers of cells (cell lines), which is based on the selective proliferation of clonal cells. PDXs keep the histological features, genomic signatures and genetic heterogeneity of cells in a tumor mass [45]. In addition, PDX tumors provide enough material to also perform phosphoproteomics. However, PDX samples have limitations that must be considered prior to data analysis: it can take up to 6 months to generate PDX and these can be highly contaminated with mouse cells (due to sample preparation). Here, several of the significantly regulated proteins and proteins containing an alternate variant peptide were also identified within the FFPE materials. This highlights the relatively good correlation between PDX and FFPE materials and validates the use of PDX within proteogenomic applications.

To identify regulated proteins and phosphorylated sites in patient-specific tumor samples, we performed comparisons against healthy melanocyte cells. This experimental rationale was required due to the lack of matching patient-specific healthy tissue samples. While the proteome profile of this melanocyte sample is not patient-specific, it acts as a healthy standard and allows patient-to-patient comparability. While still uncommon, future global proteogenomic studies will benefit from this experimental design which compares patient-specific tumors to normal tissues [46], as it best recapitulates patient-to-patient heterogeneity and the tumor’s microenvironment [47,48]. Here, a comparison of tumor cells against melanocytes highlighted several over-represented signaling pathways based on significantly regulated proteins. Interestingly, the down-regulated proteins in patient IDs 101 and 110 against melanocytes were over-represented for “insulin growth factor signaling”; whereas the down-regulated proteins in both ICi-treated patients (IDs 111 and 129) against melanocytes resulted in an over-representation of the “AURKA interaction” pathway. Several pathways were found to be over-represented based on the up-regulated proteins between patients and melanocytes. For example, “interleukin-9 signaling” for patient ID 101, “NLRP1 inflammasome” for patient ID 101 and 111, “ENCAM1 interaction” for patient 110 and “WNT signaling” for patient ID 129. These pathways are involved in melanoma and could be linked to immunotherapy [49,50]. In addition, we performed a pathway over-representation analysis of proteins containing alternate variant peptides and identified several known mechanisms involved in the response to immunotherapy including “mTOR and PI3K-AKT signaling”, “signaling by TLR4 cascade” and “activation of IFN production” [18,51]. Interestingly, the pathway “TP53 regulates metabolic genes” was over-represented for alternate variant protein isoforms in nearly all patients (besides patient ID 101). TP53 is frequently mutated in several cancer types including melanoma and many metabolic pathways are regulated by TP53, influencing energy metabolism and the growth of cancer cells [52].

The integration of genomics, proteomics and phosphoproteomics allowed the reconstruction of the patient-specific cancerous signaling network. As reported in the literature, the number of human protein–protein interactions [53], combined with clinical knowledge [54], has considerably increased in recent years, paving the way for precision medicine. The importance of network reconstruction has been exemplified in the literature, notably to investigate network-attacking mutations [27], identify genomic alterations for therapeutic combinations [55,56] or determine novel targets from differential networks [57]. Here, the network models allowed the prediction and prioritization of several drugs based on their potential to disrupt the perturbed signaling network of each patient. Interestingly, ten drugs were predicted to be in common to all patient IDs. These drugs could be potentially used for drug-treatment in melanoma, such as tromethamine that inhibits amyloid beta A4 protein and is already in use in a number of cancers [58]. For patient ID 110, we were able to generate a sample-specific cell line and experimentally validate some of the predicted drugs. Notably, we observed a reduction in cell viability upon treatment with vandetanib (inhibitor of EGFR and VEGF), as well as bosutinib (inhibitor of MAP2K1 and 2). The kinase inhibitor bosutinib was previously reported to inhibit solid tumors including in the pancreas and in melanoma [59,60]. In contrast, the predicted MAP2K1 and 2 inhibitor trametinib did not show an effect on the cell viability for this cell line. Several reasons may explain the differing results of bosutinib and trametinib, for example, the variant on the target protein may influence the drug binding in the case of trametinib. Alternatively, the effect observed with bosutinib could be a result of off-target inhibition (e.g., BCR, ABL1, LYN, SRC) [61,62].

We investigated further the naïve and ICi-treated patients based on significant protein changes, including the proteins harboring the amino acid variant. A comparison of protein expression levels revealed that proteins that were down-regulated in ICi-treated cells compared to naïve cells that were involved in pathways related to the PD-1/PD-L1 and cytokine signaling. This might be due to the fact that both ICi-treated models were isolated from metastases that had grown upon anti-PD-1 therapy and thereby can be considered as resistant lesions. Lack of PD-L1 expression as well as a loss of IFNg signaling are long-known resistance mechanisms to ICi therapy [18,63]. This analysis also revealed that 86 significantly regulated proteins harbored an alternate variant peptide, which revealed an over-representation of the pathways important for melanoma development and immune response. This can be explained in several ways, for example these variants could accumulate in the corresponding pathways and provide a survival advantage for cancer cells. Alternatively, the proteins harboring these variants have intrinsic characteristics that facilitate their detection by MS (e.g., protein abundance, protein length), thus facilitating the detection of amino acid variants [64,65].

## 4. Materials and Methods

Skin metastases were collected during surgery and compared to blood. In total, we analyzed four metastatic tumors and melanocytes as a control. In addition, primary tissues were injected into mice to obtain patient-derived xenografts (PDX). The use of human tissue from an internal biobank was approved by the Local Research Ethics Committee (IEC) Tuebingen (781/2018BO2) and experiments were performed in accordance with the declaration of Helsinki Principles.

### 4.1. Generation of Patient-Derived Xenografts

To generate patient derived xenografts (PDX), tumor tissue was finely minced using the cross-blade technique, digested in nevi solution (HBBS (w/o Ca^2+^ and Mg^2+^) with 0.05% collagenase, 0.1% hyaluronidase and 0.15% dispase) and filtered through a 100 µm cell strainer. The melanoma cell suspension was implanted with Matrigel (Corning Life Sciences, Lowell, MA, USA) subcutaneously in NSG (NOD.Cg-Prkdc^scid^ Il2rg^tm1Wjl^/SzJ) mice, leading to patient-derived xenografts. Tumor grafts were harvested when they reached a diameter of 10 to 15 mm, digested as above, resuspended in Biofreeze medium (Biochrom/Merck, Berlin, Germany) and 1 mL per cryotube of the cell suspension was frozen for short-term cryoconservation in −80 °C and for long-term storage in liquid nitrogen.

### 4.2. Generation of Primary Human Melanoma Cell Lines

Patient-derived cells were acquired directly from tumor tissue from patients. The tissue was cut into small pieces and incubated for 1 h in an enzyme mix of collagenase, hyaluronidase and trypsin at 37 °C. To stop the digestion process, a cell culture medium was added. The solution was thoroughly mixed by pipetting up and down and was finally filtered through a cell strainer (100 µm). By centrifugation at 1200× *g* for 5 min, the cells were pelleted and resuspended in fresh cell culture media.

### 4.3. Isolation and Cultivation of Melanocytes and Fibroblasts

Primary human melanocytes and fibroblasts were isolated out of foreskin according to the protocol of CELLnTEC (CELLnTEC Advanced Cell Systems AG, Bern, Switzerland). After isolation, cells were cultured in a CnT-40 (melanocytes) or CNT-PR-F (fibroblasts) medium containing antibiotics/antimycotics (CnT-GAB10 or CnT-ABM10).

### 4.4. Protein Extraction from Patient-Derived Xenografts

Cell lysis of snap-frozen patient-derived xenografts (PDX) was performed with a lysis buffer (6 M urea, 2 M thiourea, 10 mM Tris-HCl pH 8.0) supplemented with a protease inhibitor (complete Mini EDTA-free tablets; Roche, Basel, Switzerland) and phosphatase inhibitor buffers (5 mM glycerol-2-phosphate, 5 mM sodium fluoride, and 1 mM sodium orthovanadate). Glass beads (zirconia/glass beads 0.23 mm; Carl Roth GmbH, Karlruhe, Germany) were added and a cell lysis was performed in a BeadBug microtube homogenizer (3 cycles, 1 min at full speed; Sigma-Aldrich, St. Louis, MO, USA). Cell extracts were centrifuged at 13,000 rpm for 20 min and proteins were purified by acetone precipitation. Briefly, cell lysates were mixed with 8 volumes of ice-cold acetone and one volume of methanol and incubated overnight at −21°C. After centrifugation (2800× *g*, 20 min, 10 °C), protein pellets were washed with 80% acetone and dissolved in a lysis buffer. The protein concentration was determined by a Bradford assay.

### 4.5. Protein Extraction from Melanocytes

Cells were washed twice with PBS and a cell lysis was performed with a lysis buffer (6 M urea, 2 M thiourea, 10 mM Tris-HCl pH 8.0) supplemented with a protease inhibitor (complete Mini EDTA-free tablets, Roche), phosphatase inhibitor buffers (5 mM glycerol-2-phosphate, 5 mM sodium fluoride, and 1 mM sodium orthovanadate) and 1% *N*-Ocetylglucoside (NOG) on ice for 10 min. DNA and RNA was removed with benzonase (Merck, Darmstadt, Germany) for 10 min at RT followed by centrifugation at 2800× *g* (10 °C, 20 min). Proteins were purified by acetone precipitation and the protein concentration was determined by a Bradford assay.

### 4.6. Protein Extraction from Formalin-Fixed Paraffin Embedded Tissue Preparation

Formalin-fixed paraffin-embedded (FFPE) tumor tissues were first de-paraffinized by two washes in xylene (5 min, 50 °C) followed by three serial washes in ethanol (100%, 95% to 70%) for 10 min each. Ethanol was removed completely and sections were air-dried. Lysis was carried out in 4% (*v*/*v*) SDS, 50 mM DTT, 100 mM HEPES with a pH of 7.5 supplemented with a protease inhibitor at 95 °C for 60 min and by sonication for 15 min. Proteins were purified by acetone precipitation and the protein concentration was determined by a Bradford assay.

### 4.7. Sample Preparation for MS Analysis

Purified protein pellets of different sample types were dissolved in a lysis buffer (6 M urea, 2 M thiourea, 10 mM Tris-HCl pH 8.0), reduced using 100 mM DTT and alkylated using 50 mM iodoacetamide followed by pre-digestion using endopeptidase Lys-C (Lysyl Endopeptidase; Wako Chemicals, Richmond, VA, USA) for 3 h. After diluting the sample to 2 M Urea with 10 mM ammonium bicarbonate, proteins were digested into peptides using sequencing grade trypsin (1 µg per 100 mg protein; Promega Corporation, Madison, WI, USA) overnight. Peptides were then acidified with 1% TFA and then either purified on C18 stage tips (as described previously) or purified on Sep-Pak C18 Cartridge (Waters) and eluted in 80% ACN for high pH reverse phase chromatography.

### 4.8. High-pH Reverse Phase Chromatography of PDX and Melanocyte Samples

High pH reverse phase chromatography was conducted using an Ultimate 3000 HPLC (Thermo Fischer Scientific, Waltham, MA, USA) equipped with xBridge BEH130 C_18_ 130A, 3.5 µm, 4.6 × 250 mm column (Waters, Milford, MA, USA) as described previously [33]. In brief, peptides were eluted with an 80 min gradient generated from solvent A (5 mM NH_4_OH) and solvent B (5 mM NH_4_OH, 90% ACN) at pH 10. Fractions were collected in the first 60 min of the gradient and concatenated into 30 pools followed by vacuum centrifugation. Peptide pools were resuspended in 500 µL 80% ACN, 10 µg of the pool was concentrated and desalted on StageTips prior to the LC-MS/MS measurements for proteome analysis.

### 4.9. Phosphopeptide Enrichment

Phosphopeptides were enriched using TiO_2_ beads (Titansphere, 10 µm; GL Sciences, Shinjuku-Ku, Japan) as described previously [66]. A total of 1 mg of beads (in 80% ACN, 1% TFA) were added to acidified high pH fractions and incubated for 30 min in a rotation wheel. Phosphopeptide-bound TiO_2_ beads were sequentially washed with 30% ACN, 1% TFA, followed by 50% ACN, 1%TFA and 80% ACN, 1% TFA Peptides were eluted with 5% NH_4_OH into 20% TFA followed by 80% ACN in 1% FA. The eluate was reduced by vacuum centrifugation, the pH was adjusted to <2.7 with TFA and the peptides were desalted on C18 StageTips.

### 4.10. Liquid Chromatography–Mass Spectrometry

LC–MS/MS runs were performed on EASY-nLC 1200 UHPLC (Thermo Scientific) coupled to Q Exactive HF and HFX Orbitrap mass spectrometers (Thermo Scientific). The peptides were separated on 20 cm analytical HPLC columns (75 μm ID PicoTip fused silica emitter (New Objective, Berks, UK); in-house packed using ReproSil-Pur C18-AQ 1.9-μm silica beads (Dr Maisch GmbH, Ammerbuch, Germany)) using a water-acetonitrile gradient of 60 min and 90 min for proteomic samples and phosphoproteomic sample fractions, respectively. The FFPE samples were measured twice with a 60 min and 130 min gradient. Gradients were generated by solvent A (0.1% formic acid) and solvent B (80% ACN in 0.1% acetic acid) with a flow rate of 200 nL/min at 40 °C. Peptides were ionized by nanoelectrospray ionization at 2.3 kV and a capillary temperature of 275 °C. For high pH proteomic fractions and FFPE samples, each full spectrum, acquired with 60,000 resolution (automated control target of 3e6; fill time 25 ms for Q Exactive HF and 20 ms for Q Exactive HFX), was followed by 12 tandem MS (MS/MS) spectra, where the 12 most abundant multiply charged ions were selected for MS/MS sequencing with a resolution of 30,000, an automated control target of 1e5, an injection time of 45 ms and collision energy of 27% for Q Exactive HF and 28% for Q Exactive HFX. For phosphopeptide-enriched samples, full MS scans were acquired with a resolution of 60,000 (AGC target 3e6, fill time 25 ms). The seven most abundant multiply charged ions were selected for MS/MS sequencing with a resolution of 45,000 on Q Exactive HFX and 60,000 on Q Exactive HF, an AGC target of 1e5 and a fill time of 220 ms.

### 4.11. DNA Extraction and Sequencing from Blood and Snap-Frozen Primary Tissue

For patient IDs 110 and 129, genomic DNA was extracted from blood and snap-frozen primary tissue using a GeneElute mammalian genomic DNA isolation kit (Sigma-Aldrich) according to the manufacturer’s instructions with slight modifications. Human snap-frozen tissue was incubated in lysis solution C solution at 55 °C overnight, whereas blood samples were incubated for 10 min. DNA was purified on GeneElute MiniPrep columns and eluted with nuclease-free water. For patient ID 101, genomic DNA was isolated by c.ATG Core Facility in Tuebingen using the QIAamp DNA Mini (QIAGEN, Hilden, Germany) kit as recommended by the manufacturer.

At the c.ATG Core Facility in Tuebingen, the genomic DNA from each sample was assessed for quantity and quality on Nanodrop spectrophotometer (ThermoFisher Scientific), Qubit Fluorometer (ThermoFisher Scientific) and Bioanalyzer (Agilent, Santa Clara, CA, USA) instruments. The exome captures and libraries were prepared using Sureselect XT Human All Exon V7 Low Input kit (Agilent) with dual indexing according to the manufacturer’s instructions. The resulting libraries were sequenced on a NovaSeq 6000 instrument (Illumina, San Diego, CA, USA) using S2 FlowCell (200 cycles). Exome sequencing data for patient ID 111 were retrieved from the DKTK master trial [67,68].

### 4.12. Exome Sequencing Data Analysis

Raw sequence data were processed using an in-house pipeline developed at the Proteome Center, Tuebingen. The raw reads were initially quality checked using FastQC software (v. 0.11.8; Cambridge, UK) [69]. Illumina adapters and 5′/3′ low quality bases were trimmed from reads using Trimmomatic [70]. Paired-end reads from individual libraries were then aligned to the *H. sapiens* reference genome (GRCh38) using the HiSAT2 aligner [71]. Reads resulting from PCR duplication were marked using the Picard package. Germline variants were called using the GATK HaplotypeCaller workflow, while the somatic variants were identified using the GATK Mutect2 workflow [72]. Variants were recalibrated for scores and filtered (soft-filter) using GATK (v. 4.1.2.0; Cambridge, MI, USA). SnpEff software (v. 4.3T; Detroit, MI, USA) was used to perform the annotation of detected variants [73].

### 4.13. Generation of Personalized Protein Databases for MS Analyses

To integrate the proteogenomics datasets, we used an in-house bioinformatics pipeline, which is coded entirely in the R programming language [74]. The transcript nucleotide sequences were extracted from GRCh38 *H. sapiens* genome assembly and Ensembl transcript annotation (via the BSgenome and GenomicFeatures packages). These sequences were then translated in silico (from start to first stop codon) into a reference protein sequences database (Biostrings package). The called variants, within Variant Call Format files from A375 R and A375 S, were injected into each overlapping reference transcript nucleotide sequence and then translated in silico. The resulting protein sequences were written into two FASTA files containing reference variant protein sequences and sample-specific alternate variant protein sequences.

### 4.14. Prediction of the Biological Impact of the Detected Variants

In the current study, we prioritized amino acid variants based on their impact in the context of cancer, immune-checkpoints and protein phosphorylation. For this purpose, known variant sites in cancer were retrieved from CGDS [75]. These were overlapped with A375 identified variants and classified as loss/gain of sites. A list of oncogenes and tumor suppressor genes was compiled from Cosmic, ONGene, Bushman lab and Uniprot [76,77,78], whereas a list of genes involved within the immune checkpoint was retrieved from a published study [79]. Patient-specific variants found on these genes were annotated as relevant in cancer and/or immune checkpoints. In addition, each reference/alternate variant protein sequence was annotated based on whether phosphorylation sites (S/T/Y) were lost and/or gained (IRanges package). A list of known kinase motifs was retrieved from PhosphoNetworks [80] and these motifs were searched along the reference/alternate variant protein sequences. Located kinase motifs were overlapped with the position of the variants to determine the loss/gain of the motifs. Known human phosphorylation sites were retrieved from PhosphoSitePlus and Phospho. ELM databases [81,82]. The variants identified in our study, which overlapped with known phosphorylation sites, were annotated as a loss/gain of known phosphorylation. Finally, a Levenshtein similarity score was calculated between the reference and alternate variant protein sequences, whereby alternate sequences with less than 90% similarity to their reference were flagged.

Each amino acid within the variant protein sequences was attributed a “+1” score for every overlap with an impact annotation. A summed score was then calculated for each amino acid within the alternate variant sequence and the maximum summed score was reported for that variant protein isoform. Because the score depends on the number of impacts used during the annotation, we also computed a scaled maximum score (between 0 and 1), to allow a comparison between processing. Following the computation of all impacts, each variant protein isoform was ranked to allow prioritization for follow up studies.

### 4.15. Mass Spectrometry Data Analysis

The LC–MS/MS data were searched against the PCTi *H. sapiens* reference (100,906 entries) and individualized alternate databases (101 = 43,086 entries; 110 = 44,789 entries; 111 = 28,333 entries; 129 = 39,811 entries), as well as the UniProt *H. sapiens* (release 11 December 2019; 96,788 entries) and *M. musculus* (release 11 December 2019; 63,660 entries) databases and commonly observed contaminants using the Andromeda search engine integrated into MaxQuant software (version 1.5.2.8, Munich, Germany) [83]. Carbamidomethylation of cysteine (C) was set as a fixed modification and oxidation of methionine, phosphorylation at serine, threonine or tyrosine were defined as variable modifications. Trypsin/P was selected as a protease. No more than two missed cleavages were allowed. The MS tolerance was set at 4.5 ppm and MS/MS tolerance was set at 20 ppm for the analysis using the HCD fragmentation method. The false discovery rate (FDR) for peptides and proteins was set to 1%. The PDX and FFPE samples were quantified using intensity-based absolute quantification (iBAQ).

### 4.16. Statistical Analyses and Data Visualization

Statistical analyses were performed with Perseus software suite (version 1.6.5.0, Munich, Germany). We initially filtered out all reverse and potential contaminants hits. Because PDX samples can have varying amounts of murine protein contamination (due to sample generation), we annotated each ENSEMBL protein ID with the corresponding taxonomic information (i.e., *Homos sapiens* or *Mus musculus*). Identified proteins and phosphorylation sites were divided into three classes based on taxonomic classification: class I contained proteins/sites annotated exclusively as human, class II were shared between human and mouse, and class III were annotated exclusively as mouse (see Table S2). Because proteins of murine origin were irrelevant in this study, we filtered out all proteins and phosphorylated sites of class III. A list of identified filtered protein and phosphorylation sites for each sample are provided in Tables S1–S4. To find significant differences between the PDX/FFPE samples and melanocytes, label-free quantification was used and outliers were determined for the log_2_-transformed ratios using significance B (*p*-value ≤ 0.05). The ratios of identified phosphorylation sites were normalized to the ratios of corresponding protein groups. For significantly up- and down-regulated proteins (phosphorylation sites) as well as variant protein isoforms, pathway over-representation was performed. The resources used for the annotation of proteins were Gene Ontology (GO), Biological Processes (GOBP), GO Cellular Compartment (GOCC), GO Molecular Functions (GOMF) and Kyoto Encyclopedia of Genes and Genomes (KEGG) and Reactome Pathway database (Reactome). The Fisher-Exact test (*p*-value ≤ 0.2) was used to check for over-represented categories among significantly regulated proteins and variant protein isoforms. A list of all the over-representation results is provided in Tables S2 and S4.

In order to compare naïve versus ICi-treated patients, the IBAQ intensities of naïve or ICi-treated patients were averaged and the ratio of ICi-treated versus naïve patients was calculated. Outliers were determined with Significance B (FDR ≤ 0.05) using the log_2_-transformed ratio. For significantly regulated proteins and significantly regulated proteins harboring an alternate variant peptide, hierarchical clustering was performed and a visual heat map representation of the clustered matrix was produced. Pathway over-representation and the Fisher-Exact test (*p*-value ≤ 0.2) was performed for each cluster.

Venn diagrams to show the overlap between the identified nucleotide variants from the WES analysis and between the protein identifications from the PDX and FFPE material were performed with the online tool https://www.stefanjol.nl/venny (accessed on 21 October 2021).

Protein-protein interaction networks were generated online via String (https://string-db.org/) (accessed 21 October 2021). based on significantly changing (between ICi-treated and naïve patients) alternate variant isoforms.

### 4.17. Identification of Amino Acid Variants

Our in-house proteogenomics bioinformatic pipeline was used to integrate the WES and MS datasets, specifically to check which variants were identified across omics datasets. Initially, the reference (i.e., corresponded to the amino acid sequence from the reference database) and the alternate variant (i.e., corresponded to the sequence containing an amino acid variant) protein sequences were digested in silico according to laboratory conditions, i.e., digestion with trypsin and up to two missed cleavages (cleaver package). The overlap of MS-identified peptides with in silico digested peptides led to the classification into the reference (non-mutated peptide that overlapped with the position of the variant on the reference protein), alternate (mutated peptide that overlapped with the position of the variant on the alternate protein) or unspecific (non-mutated peptide that did not overlap with any mutated positions) variant peptides. On the basis of this peptide classification, we summarized the identification of the peptides as per the variant protein isoforms, allowing coverage of the characterization into reference only, alternate only, reference and alternate or unspecific. We finally focused on PTM (as implemented in the MaxQuant processing), which here consisted of phosphorylation sites. Reference and/or alternate variant peptides found to be phosphorylated were flagged as such, as well as those where the phosphorylation occurred directly on the variant sites (either on reference or alternate variant sequences). This coverage information was exported within MaxQuant style processing results (tab-separated file as output).

We also generated interaction networks within the R programming environment [74]. These networks were generated using protein–protein (using BioGRID database), drug–target (using DrugBank database) and predicted kinase–substrate (PCTi results) interactions [53,78]. The generated networks were exported (using igraph and RCy3 packages) into Cytoscape for further customization [84].

### 4.18. Signaling Network Reconstruction

We reconstructed the network of protein–protein interactions using the BioGRID database (release 3.5.169) [53]. We used only interactions that were reported in *H. sapiens* and showed at least two types of experimental evidence (e.g., two publications, two methods). Networks were generated undirected, as such information was missing from BioGRID. In addition, self-linked interactions and orphan nodes were removed (igraph package). For the patient-specific network (comparison against melanocytes), we retrieved protein–protein interactions strictly between the significantly changing proteins or phosphorylation sites. For protein target prioritization, we ranked (from high to low) the nodes based on the number of edges within each interaction network and retained only the top 200 nodes. The patient-specific networks were exported (using igraph and RCy3 packages) into Cytoscape to improve visual formatting [84].

Possible drugs interacting with each patient-specific network were retrieved from the DrugBank database (release 24.10.2019) based on their targets [85]. Only drugs showing an effect in *H. sapiens* were used. All drugs were retained, irrespective of their category (e.g., inhibitor), chemical kingdom (e.g., organic compound) or approval status (e.g., approved, experimental). The specificities of the drugs, interacting with nodes from the generated network, were calculated based on all possible targets reported in the DrugBank database. Drugs were prioritized further by summing the number of interactions their targets had within the network.

For the network of naïve versus ICi-treated patients, we initially selected significantly changing proteins that were involved in the immune system (based on Reactome annotation). We then retrieved the interactions between the significantly changing proteins, as well as their direct interactors. Network hubs were highlighted by increasing the node size and the difference between naïve and ICi-treated patients was displayed via color-coding. Only the top 50 nodes were retained for this network. The immune-related network was exported (using igraph and RCy3 packages) into Cytoscape to improve visual formatting [84].

### 4.19. Cell Viability Assay

For the MTS viability assay, melanoma cells were seeded at a density of 2500 cells in 100 µL per well in a 96-well plate. The following day the cells were treated. The treatment was conducted via serial dilution ranging from 0.039 µM to 20 µM of the different inhibitors. As a control, wells without any treatment and wells with medium plus DMSO were used. For each treatment and cell line, quadruplicates were tested. Cells were treated for 72 h at 37 °C. Afterwards, 20 µL MTS solution (2 mg/mL) was added to each well followed by a 2 h incubation period. Absorbance was measured with a microplate reader (Tristar, Berthold) at a wavelength of 495 nm. Control wells were defined as 100% cell viability.

## 5. Conclusions

Individualized proteogenomics allows the detection of sample-specific variants at the genome, proteome and PTM levels. Here, we studied the mutational landscape of four clinical patients in response to immunotherapy. Our dataset will serve the cancer research community as a resource of clinical genomic, proteomic and phosphoproteomic profiles, which are still sparse in melanoma. Our approach revealed a patient-specific mutational landscape and their accumulation in signaling pathways, whereas network modeling predicted personalized drug interventions and highlighted differences in immune response between ICi-treated and naïve patients. While our findings will be of limited value for characterizing general pathways that cause the disease, they provide an insight into the complexity of the mutational landscapes of individual patients, they reveal the extent to which genomic variants influence proteins and their modifications and they underscore the need for a personalized approach to cancer treatment.

## Data Availability

The mass spectrometry proteomics data have been deposited to the ProteomeXchange Consortium via the PRIDE partner repository with the dataset identifier PXD028700 [86]. The WES bioinformatics pipeline is available online [87]. Excel files containing the analyzed data are provided in Supplementary Tables.

## Supplementary Materials

The following are available online at https://www.mdpi.com/article/10.3390/cancers13215411/s1; Figure S1: The mutational landscape of melanoma patients in response to immunotherapy; Figure S2: Comparison of tumor cells against melanocytes highlights patient-specific signaling pathways; Figure S3: Integration of genomics, proteomics and drug database prioritizes actionable targets; Figure S4: Differential protein expression between naïve and ICi-treated patients; Table S1: The mutational landscape of melanoma patients in response to immunotherapy; Table S2: Comparison of tumor cells against melanocytes highlights patient-specific signaling pathways; Table S3: Integration of genomics, proteomics and drug database prioritizes actionable targets; Table S4: Differential protein expression between naïve and ICi-treated patients.
