# Supplementary material for: Individualized Proteogenomics Reveals the Mutational Landscape of Melanoma Patients in Response to Immunotherapy
Source: Cancers (Basel). 2021 Oct 28;13(21):5411. doi: 10.3390/cancers13215411 (PMC8582461; doi:10.3390/cancers13215411)
Supplement: Supplementary file 1 [file cancers-13-05411-s001.zip › cancers-1421686 Supplementary Figures.pdf]

cancers

MDPI

Supplementary Macterial: Individualized Proteogenomics Reveals the Mutational Landscape of Melanoma Patients in Response to Immunotherapy

Marisa Schmitt, Tobias Sinnberg, Heike Niessner, Andrea Forschner, Claus Garbe, Boris Macek and Nicolas C. Nalpas

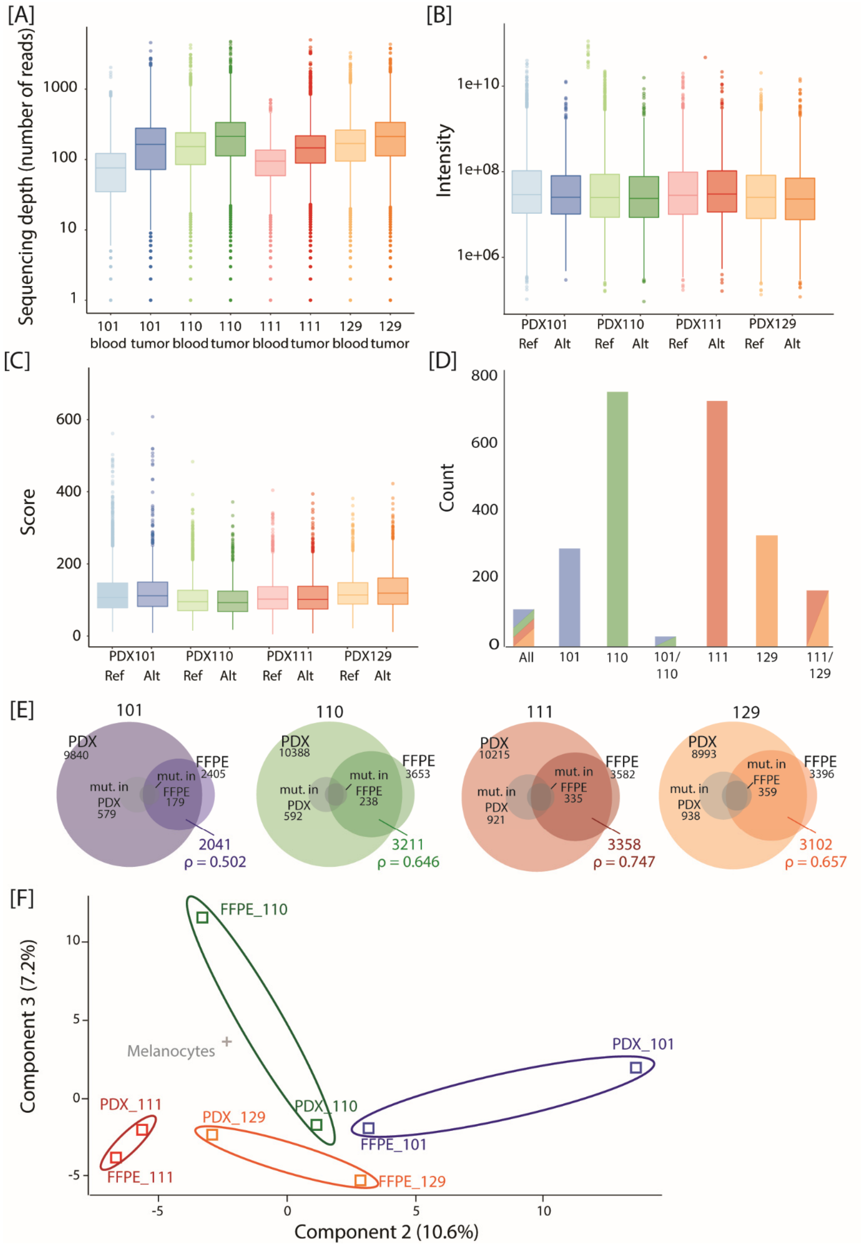

Figure S1. The mutational landscape of melanoma patients in response to immunotherapy. [A] WES sequencing depth for each patient ID and sample type. [B] MS-measured intensity of identified reference (Ref) and alternate (Alt) variant peptides for each patient ID. [C] The MaxQuant score of reference (Ref) and alternate (Alt) variant peptides identified by MS for each patient ID. [D] The number of sample-specific alternate variant peptides that are identified by MS for each patient ID. [E] Overlap of quantified protein groups and proteins harboring alternate variant peptides (mut.) between PDX and FFPE sample type for each patient ID. [F] Principal component analysis using protein abundances shows the separation of patients, as well as samples types (PDX and FFPE). Clustering of samples from same patient are indicated by colored ellipses, with blue (patient ID 101), green (patient ID 110), red (patient ID 111) and orange (patient ID 129).

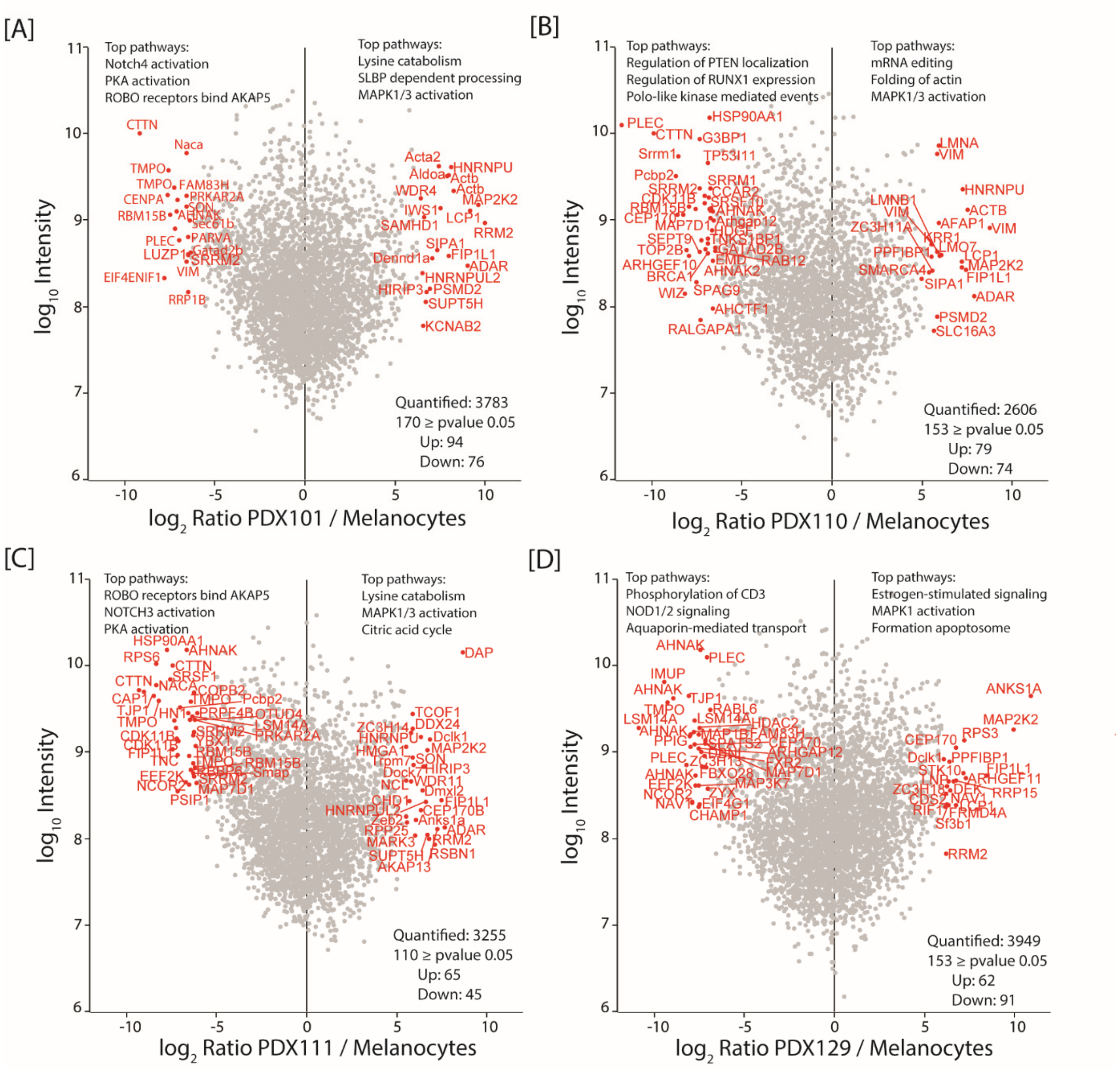

Figure S2. Comparison of tumor cells against melanocytes highlights patient-specific signaling pathways. Phosphorylation sites were quantified in PDX samples versus melanocytes for patient ID 101 [A], 110 [B], 111 [C] and 129 [D]. Significant up-or down-regulated phosphorylation sites are marked in red (significance B, p-value ≤ 0.05). Results for over-represented Reactome pathways based on significant up-or down regulated phosphorylation sites are depicted in the upper part of each panel (Fisher-Exact test, p-value ≤ 0.2).

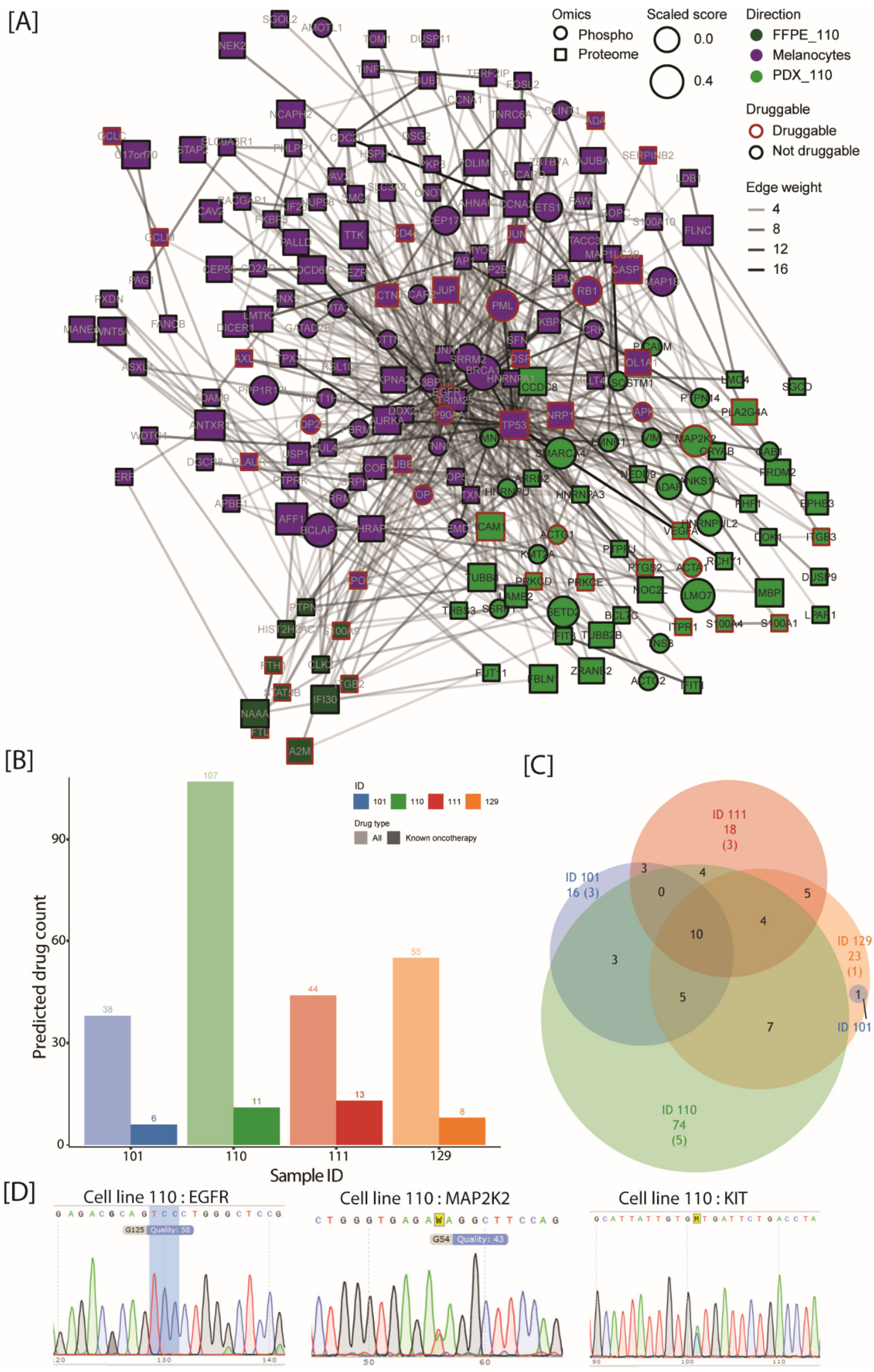

Figure S3. Integration of genomics, proteomics and drug database prioritizes actionable targets. [A] The interaction signaling network for patient ID 110 is generated based on list of significantly regulated proteins (square) and phosphorylation sites (circle). Only the top 200 entries are displayed (ranked based on their interaction degree). Entries are colored based on whether they are up-regulated in PDX (light green), FFPE (dark green) or melanocytes (purple). Entries that can be targeted by a drug are displayed with a red stroke. Entries were prioritized based on their importance in context of melanoma and immune checkpoint inhibition therapies (from 0 = no impact, up to 1 = high impact) and their node size increased accordingly. [B] The number of predicted sample-specific drugs for each patient ID. Lighter colors are indicative of all predicted drugs, whereas darker colors correspond to drugs previously used in cancer therapies. [C] Overlap of predicted drugs for each patient ID. Numbers not in bracket represent the overlap of all predicted drugs, whereas number in bracket indicate the overlap of known oncotherapies. [D] Sanger sequencing result for targets (EGFR, MAP2K2 and KIT) used for cell viability assay for cell line 110.

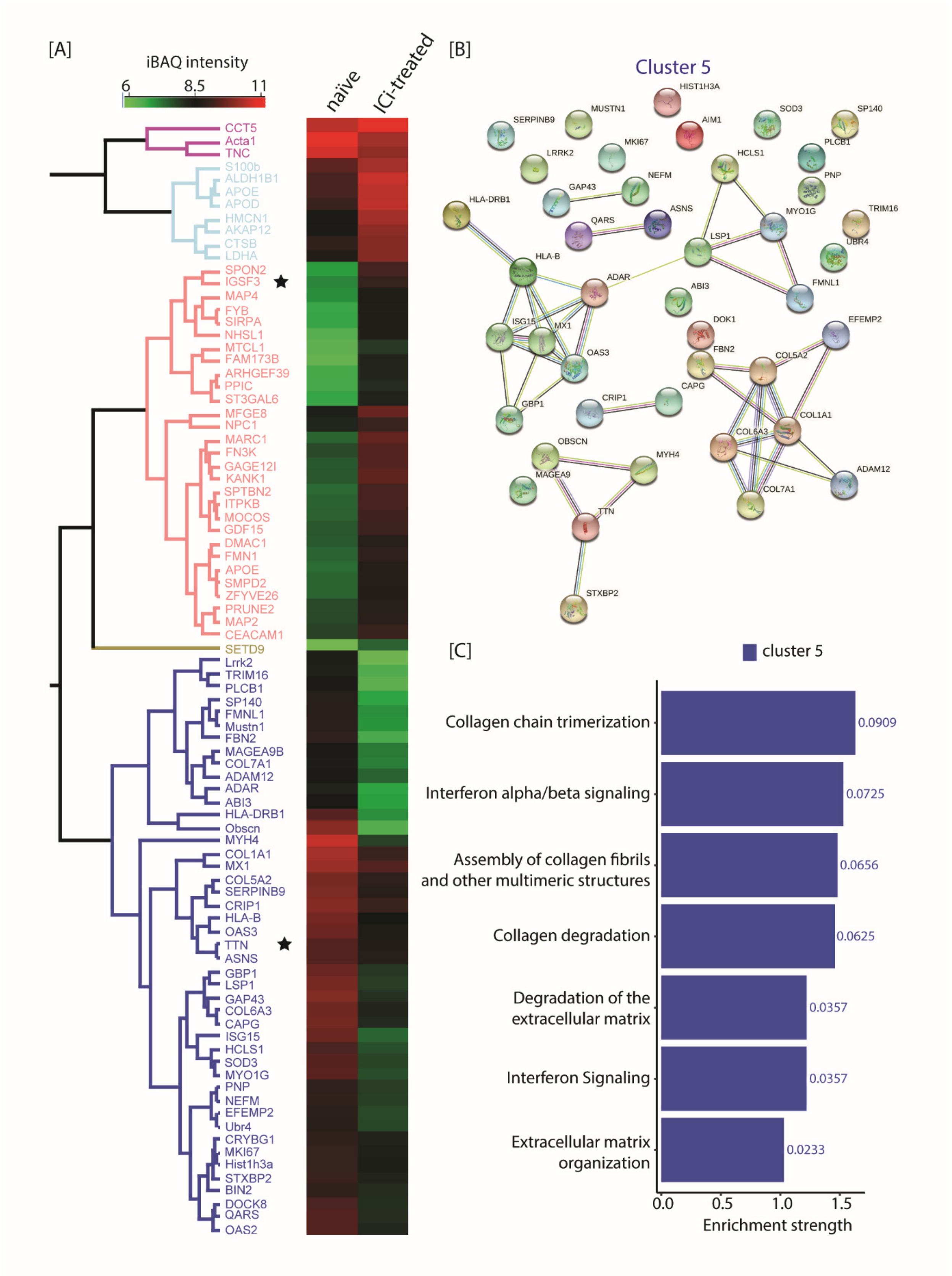

Figure S4. Differential protein expression between naïve and ICi-treated patients. [A] Heat map of significantly regulated proteins containing alternate variant peptides between naïve and ICi-treated patients (Sig. B, FDR ≤ 0.05). Color code depicts $\log_{10}$ transformed IBAQ intensities of proteins. Proteins harboring an alternate variant peptide identified to be phosphorylated on variant site are marked with a star. [B] Proteins of cluster 5 (dark blue) were mapped to string functional protein association networks. [C] String pathway analysis of proteins in cluster 5. The count of the observed proteins divided by the count of the background protein within the pathway is represented in the enrichment strength. Text on the right of the bar correspond to the String pathway analysis score.

Table S1–S4 can be found in separate Excel files.
